# A Longitudinal Follow-up of Autoimmune Polyendocrine Syndrome Type 1

**DOI:** 10.1210/jc.2016-1821

**Published:** 2016-06-02

**Authors:** Øyvind Bruserud, Bergithe E. Oftedal, Nils Landegren, Martina M. Erichsen, Eirik Bratland, Kari Lima, Anders P. Jørgensen, Anne G. Myhre, Johan Svartberg, Kristian J. Fougner, Åsne Bakke, Bjørn G. Nedrebø, Bjarne Mella, Lars Breivik, Marte K. Viken, Per M. Knappskog, Mihaela C. Marthinussen, Kristian Løvås, Olle Kämpe, Anette B. Wolff, Eystein S. Husebye

**Affiliations:** Department of Clinical Science (Ø.B., B.E.O., E.B., B.G.N., L.B., P.M.K., K.Lo., A.B.W., E.S.H.), University of Bergen, 5021 Bergen, Norway; Department of Medicine (Solna) (N.L., O.K.), Karolinska Institutet, 171 76 Stockholm, Sweden; Science for Life Laboratory (N.L.), Department of Medical Sciences, University of Uppsala, 751 05 Uppsala, Sweden; Department of Medicine (M.M.E., K.Lo., E.S.H.), Haukeland University Hospital, 5021 Bergen, Norway; Department of Medicine (K.Li.,), Akershus University Hospital, 1474 Nordbyhagen, Norway; Department of Endocrinology (K.Li., A.P.J.), Oslo University Hospital, 0372 Oslo, Norway; Department of Pediatrics (A.G.M.), Oslo University Hospital, 0424 Oslo, Norway; Division of Internal Medicine (J.S.), University Hospital of North Norway, 9019 Tromsø, Norway; Institute of Clinical Medicine (J.S.), University of Tromsø, The Artic University of Norway, 9019 Tromsø, Norway; Department of Endocrinology (K.J.F.), St. Olavs Hospital, 7006 Trondheim, Norway; Department of Medicine (Å.B.), Stavanger University Hospital, 4011 Stavanger, Norway; Department of Medicine (B.G.N.), Haugesund Hospital, 5504 Haugesund, Norway; Department of Medicine (B.M.), Østfold Hospital, 1603 Fredrikstad, Norway; Department of Immunology (M.K.V.), Oslo University Hospital, 0372 Oslo, Norway; University of Oslo (M.K.V.), 0372 Oslo, Norway; Center for Medical Genetics and Molecular Medicine (P.M.K.), Haukeland University Hospital, 5021 Bergen, Norway; Department of Clinical Dentistry (M.C.M.), Faculty of Medicine and Dentistry, University of Bergen, 5021 Bergen, Norway; and Oral Health Centre of Expertise in Western Norway (M.C.M.), 5021 Bergen, Norway

## Abstract

**Context:**

Autoimmune polyendocrine syndrome type 1 (APS1) is a childhood-onset monogenic disease defined by the presence of two of the three major components: hypoparathyroidism, primary adrenocortical insufficiency, and chronic mucocutaneous candidiasis (CMC). Information on longitudinal follow-up of APS1 is sparse.

**Objective:**

To describe the phenotypes of APS1 and correlate the clinical features with autoantibody profiles and autoimmune regulator (*AIRE)* mutations during extended follow-up (1996–2016).

**Patients:**

All known Norwegian patients with APS1.

**Results:**

Fifty-two patients from 34 families were identified. The majority presented with one of the major disease components during childhood. Enamel hypoplasia, hypoparathyroidism, and CMC were the most frequent components. With age, most patients presented three to five disease manifestations, although some had milder phenotypes diagnosed in adulthood. Fifteen of the patients died during follow-up (median age at death, 34 years) or were deceased siblings with a high probability of undisclosed APS1. All except three had interferon-ω) autoantibodies, and all had organ-specific autoantibodies. The most common *AIRE* mutation was c.967_979del13, found in homozygosity in 15 patients. A mild phenotype was associated with the splice mutation c.879+1G>A. Primary adrenocortical insufficiency and type 1 diabetes were associated with protective human leucocyte antigen genotypes.

**Conclusions:**

Multiple presumable autoimmune manifestations, in particular hypoparathyroidism, CMC, and enamel hypoplasia, should prompt further diagnostic workup using autoantibody analyses (eg, interferon-ω) and *AIRE* sequencing to reveal APS1, even in adults. Treatment is complicated, and mortality is high. Structured follow-up should be performed in a specialized center.

## Abstract (precis)

Extended follow-up (1996–2016) of 52 Norwegian APS-1 patients describing clinical manifestations, natural course including mortality, autoantibody profiles and Autoimmune Regulator (AIRE) mutations.

Autoimmune polyendocrine syndrome type 1 (APS1) is a monogenic disease, also known as autoimmune polyendocrinopathy-candidiasis-ectodermal dystrophy (OMIM no. 240300). Clinically, APS1 is defined by the presence of two of the three major components: hypoparathyroidism, primary adrenocortical insufficiency (PAI), and chronic mucocutaneous candidiasis (CMC) (1). However, the syndrome also includes many less known disease components, and the clinical presentation is highly variable (2). One major manifestation combined with a sibling with APS1 also qualifies for the diagnosis. The disease usually presents in childhood and adolescence, but many patients are not diagnosed until adulthood or not at all (3). APS1 patients have an increased risk of cancer and increased mortality compared with the general population (4). The diagnosis can also be made by finding two disease-causing mutations in the autoimmune regulator (*AIRE*) gene together with clinical manifestations (5, 6). About 115 mutations have been reported so far (7). AIRE is almost exclusively expressed in the thymus (8) and plays a crucial role in negative selection of self-reactive T cells and development of regulatory T cells (9, 10).

The highest prevalence is found among Persian Jews (1:9000) (11), Sardinians (1:14 000) (12), and Finns (1:25 000) (1). The prevalence in Norway was previously reported at 1:90 000 (3). Recently, patients with monoallelic *AIRE* mutations with dominant inheritance, characterized by a later disease onset and often milder phenotypes, were reported (13). These nonclassical forms may be much more prevalent because monoallelic *AIRE* mutations have a prevalence in the general population of about 1:1000 (13).

Most patients have autoantibodies against autoantigens expressed in the affected tissue (14), eg, the steroidogenic enzymes 21-hydroxylase (21OH) in the adrenal cortex and side-chain-cleavage enzyme (SCC) in the gonads and adrenal cortex. Recently, several novel autoantigens have been identified using proteome arrays, including the prostate-specific enzyme transglutaminase 4 (TGM4) associated with male infertility and prostatitis in *Aire*-knockout mice (15, 16). Other autoantigens identified using this technique include protein disulfide isomerase-like testis expressed (PDILT) and melanoma antigen B2 (MAGEB2), which are both expressed in testicular germ cells (17). In addition, almost all patients display autoantibodies to interferons (IFNs) and interleukins (ILs) (18, 19); they typically appear years before the corresponding clinical symptoms. Mutational analysis and assay of anti-IFN-ω autoantibodies are suggested as diagnostic options (20).

Information on longitudinal follow-up of APS1 patients is sparse, with only a few series published (21–23). Building on previous surveys of the Norwegian cohort and our National Registry of Autoimmune Diseases (3, 24), we here provide a longitudinal follow-up of the Norwegian cohort spanning two decades and presenting the natural course including mortality, autoantibody profiles, and correlations to genotype.

## Patients and Methods

### Patients

Patients were recruited from departments of medicine and pediatrics from hospitals in Norway and were included in our National Registry of Autoimmune Diseases initiated in 1996 (3). All fulfilled the diagnostic criteria for APS1 given above. The Regional Committee for Medical and Health Research Ethics approved the study, and all participants gave informed consent.

### Definitions and clinical data

The patients were assessed at least annually, including hormonal status and autoantibody profiles. All patients alive were screened for *AIRE* mutations. A dental and oral examination was performed in 31 patients, and most patients underwent esophagogastroduodenoscopy, chest x-ray, and imaging of the spleen and kidneys. Endocrinopathies were diagnosed as previously described (1). The diagnostic criteria for other disease manifestations are given in Supplemental Table 1.

### Autoantibody assays

Autoantibodies against 21OH, 17-α-hydroxylase (17OH), aromatic L-amino acid decarboxylase (AADC), glutamic acid decarboxylase 65-kDA isoform (GAD65), IFN-ω, IL-17, IL-22, MAGEB2, NACHT leucine-rich-repeat protein 5, PDILT, putative potassium channel regulator, SCC, sex-determining region Y-box 10, TGM4, tryptophan hydroxylase 1, and tyrosine hydroxylase were assayed by radio-binding ligand assay as described previously (15, 16, 25). All autoantibody assays were performed in our laboratory, and sera spanning a time period were analyzed in the same experiment to avoid between-assay variations in the indices. Parietal cell antigen autoantibodies were assayed by ELISA (Euroimmun).

### Mutational analysis of the *AIRE* gene

DNA sequencing of *AIRE* spanning the exon-intron boundaries was performed using standard methods. Primer sequences are available upon request. Copy number analysis was performed by duplex TaqMan real-time PCR as previously described (26).

### Human leukocyte antigen allele typing

The sequence-based human leukocyte antigen (HLA) genotyping was performed using SBT Resolver and Assign Software (Conexio Genomics).

### Statistical analyses

Fischer's exact test with two-sided significance performed in a 2 × 2 contingency table was used (IBM SPSS Statistics 22), testing each autoantibody against the presence of different disease components. Similarly, the associations between phenotypes, *AIRE* mutations, and different HLA alleles were tested. Specifically, we also investigated the correlation between PAI in APS1 patients and HLA class II risk genotypes for PAI in the general population as defined previously (27).

## Results

### APS1 patients ascertained

Applying our diagnostic criteria and the National Registry of Autoimmune Diseases, we included 52 individuals (28 males, 24 females) from 34 families, including three patients (Supplemental Table 2, family XXIX, patients no. 45–47) with monoallelic *AIRE* PHD1 mutations (Table 1). These patients represented all known Norwegian APS1 patients. Fifteen patients died during the follow-up period, including seven who were identified only after their death. Most of the patients participated in two earlier surveys (3, 24), but 12 identified after 2007 (3) were added to the current survey.

**Table 1.** Basic Demographics of the 52 Norwegian APS1 Patients

No. of females/males	24/28
No. of families	34
Age at onset of first component, y	0–43 (median, 8.5)
Age at death (n = 15), y	3–64 (median, 34)
*AIRE* mutations found (alleles, n = 92)	92% (85/92)
Autoantibodies (n = 45)	
Organ specific	100% (45/45)
IFN-ω	93% (42/45)

### Clinical manifestations and the classic triad

**Figure 1.**
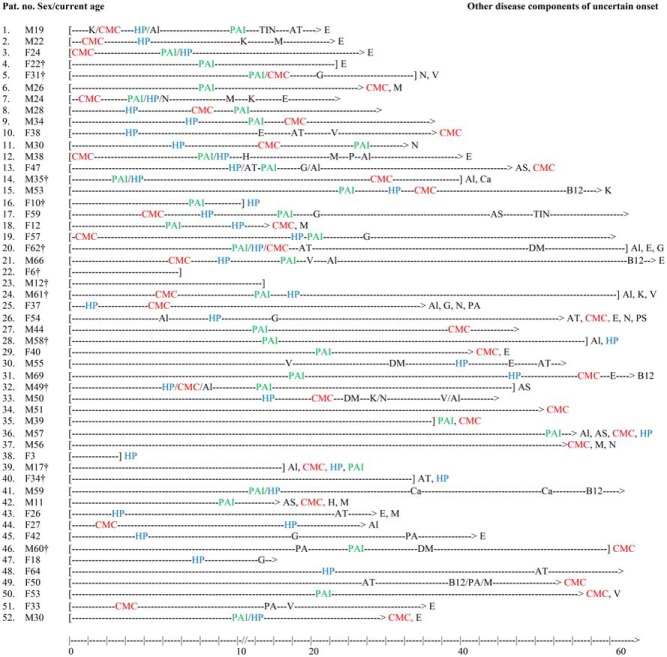
Disease histories of Norwegian APS1 patients. The lines start at birth and end at death (bracket) or at current age (>). Age at appearance of disease component is indicated by a symbol of the disease. Disease components of uncertain time of onset are listed at the end of the line. The major disease components CMC, HP, and PAI are marked in red, blue, and green, respectively. Al, alopecia; AS, asplenia; AT, hypothyroidism; Ca, cancer; DM, diabetes mellitus; E, enamel hypoplasia/defects; G, hypogonadism; H, hepatitis; HP, hypoparathyroidism; K, keratoconjunctivitis; M, malabsorption; N, nail dystrophy; P, pancreas failure exocrine; PA, pernicious anemia; PS, psoriasis; TIN, tubule interstinal nephritis; V, vitiligo.

The clinical picture was highly variable, even among members of the same family. An overview of the prevalence of the most common disease components, including sex distribution and age at presentation, is given in Table 2 and Supplemental Table 2. The most common initial manifestations were hypoparathyroidism (17 patients, 32%) and CMC (13 patients, 25%), but other components occasionally presented first. Among patients with all three major components, 14 (67%) had developed the triad by 25 years of age. In general, the disease components increased in prevalence by age, although the time courses differed markedly (Figure 1). The median number of disease components was five (range, one to eight) (Supplemental Figure 1).

**Table 2.** Prevalence of the Most Common Disease Components, Gender, and Age at Onset (n = 52)

Disease Components	Prevalence, % (n/Total n)	Females/Males, n	Median (Range) Age at Onset, y
Classic triad			
Hypoparathyroidism	73 (38/52)	18/20	9 (1–60)
PAI	63 (33/52)	11/22	13 (4–55)
CMC	77 (40/52)	15/25	7,5 (0–64)
All three	40 (21/52)	6/15	14 (4–64)
Other endocrine disorders			
Gonadal failure	33 (8/24)^a^	8/0	18 (15–25)
Diabetes mellitus	8 (4/52)	1/3	33 (23–54)
Hypothyroidism	19 (10/52)	8/2	22 (13–51)
Skin disorders			
Alopecia	31 (16/52)	5/11	19 (4–41)
Vitiligo	15 (8/52)	4/4	20 (15–51)
Gastrointestinal disorders			
Pernicious anemia or vitamin B12 deficiency	15 (8/52)	4/4	38 (13–63)
Malabsorption	23 (12/52)	5/7	21 (10–39)
Autoimmune hepatitis	4 (2/52)	0/2	5,5 (0–11)
Eye disorders			
Keratoconjunctivitis	12 (6/52)	1/5	22 (11–25)
Others			
Enamel hypoplasia	72 (18/25)^b^	9/9	
Nail dystrophy	13 (7/52)	3/4	
Asplenia	16 (5/31)	2/3	

a Percentage of female patients.

b Percentage of patients examined by dentist.

Two-thirds of the patients with hypoparathyroidism were diagnosed before the age of 15 years. One young female patient (patient 38) died of seizures at age 3 years, probably from undiagnosed hypoparathyroidism.

Thirty patients with PAI (91%) were diagnosed before age 25 years, including 21 (64%) before the age of 15 years. Two young females died in acute adrenal failure during the follow-up period (Supplemental Table 3).

The clinical course of CMC varied from periodic to chronic. Fourteen patients (27%) developed CMC before age 10 years and only three after the age of 30 years. Eleven had angular cheilitis at the time of examination and another 10 reported previous episodes. Eight patients (15%) were diagnosed with candida esophagitis, sometimes without typical symptoms or coexisting oral candidiasis. One patient (patient 34) developed stenosis of the esophagus requiring endoscopic dilation. Twelve of the 31 patients examined by a dentist tested positive for *Candida albicans* by culture. One patient (patient 51) suffered from severe candida otitis.

### Other endocrinopathies

Hypothyroidism was the third most frequent endocrinopathy followed by gonadal failure. No male hypogonadism was found. Diabetes mellitus type 1 was rare (n = 4) and had a relatively late onset.

### Oral cavity and teeth

**Figure 2.**
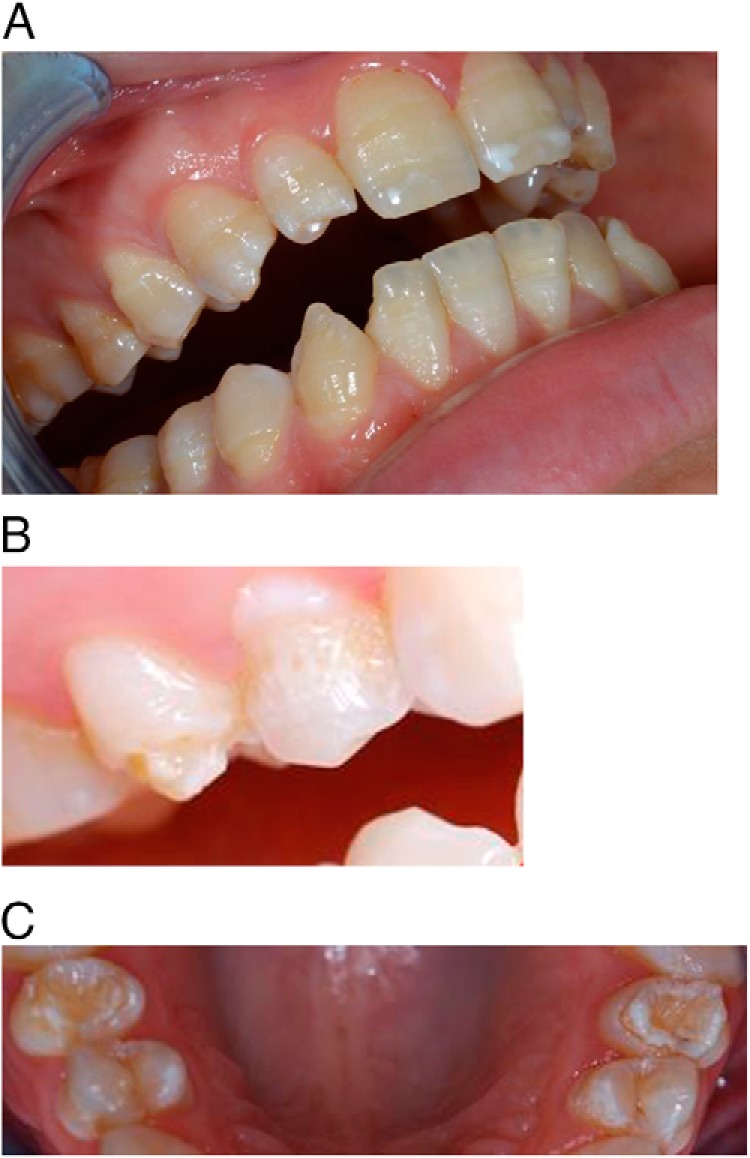
Typical enamel hypoplasia in APS1. A, Hypomineralization (white areas on front teeth) and enamel hypoplasia revealed by horizontal hypoplastic bands. B, Severe enamel hypoplasia with loss of normal enamel structure. C, Enamel hypoplasia varying in size and location, affecting both front teeth and molars.

Six of 31 examined patients had extensive composite dental restorations, most probably secondary to underlying enamel defects. Another 18 (72%) had enamel hypoplasia typical for APS1 (Figure 2), and five had enamel hypomineralization without enamel hypoplasia, which could be of different etiology. The enamel hypoplasia varied in extent and location. Nine patients had gingivitis, and eight patients presented pathologically low (below 0.1 mL/min) unstimulated salivary flow.

### Skin diseases

Vitiligo was variable in extent and location, from spots to almost universal, but was not classified into segmental or nonsegmental forms. The time of diagnosis of alopecia was highly variable, and the clinical picture varied from chronic to periodical.

### Gastrointestinal manifestations

Chronic diarrhea was interpreted as malabsorption. Pernicious anemia and vitamin B12 deficiency typically presented late but were also seen in some young individuals (patient 51). Autoimmune hepatitis was found in two young male patients (patients 12 and 42). Generally, the gastrointestinal manifestations were of variable intensity and duration.

### Ocular disease

Three patients had keratoconjunctivitis, two iridocyclitis, and one blepharoconjunctivitis. A female patient (patient 43) was diagnosed with optic neuritis at the age of 22 years.

### Other manifestations

Two patients were diagnosed with tubulointersitial nephritis. Patient 1 was diagnosed with IgA nephritis at age 15 years, and patient 17 presented with nephrotic syndrome at age 48 years. Biopsy showed local segmental nephrosclerosis. Her kidney disease is now stable after steroid treatment. Deafness was found in one female patient (patient 51).

### The natural course

Most patients follow the classic course already described for APS1: the first disease component, often one of the classic triad components, presented in childhood, with additional disease components occurring at different time intervals (Figure 1 and Supplemental Table 2). However, only 14 of 21 patients developed the full triad before the age of 25 years. Early onset was associated with a more severe phenotype, and the disease components increased in prevalence with age. Hypoparathyroidism, PAI, CMC, and autoimmune hepatitis appeared early, whereas hypothyroidism, B12 deficiency, and pernicious anemia mainly had a late onset. Furthermore, the vast majority of patients had enamel defects or hypoplasia, probably with onset in adolescence. Atypical late presentations and long intervals between components contributed to delayed diagnosis (patients 30, 31, and 46).

### Mortality and cancer

Fifteen patients died during the follow-up period, and seven patients were identified after their death (Supplemental Table 3). The major causes of death were malignant disease and adrenal and hypocalcemic crises. The median age at death was 34 years. Supplemental Table 4 gives an overview of the different malignant conditions found.

### Distribution of autoantibodies

We assayed a large panel of autoantibodies related to APS1, including the recently identified autoantigens TGM4, PDILT, and MAGEB2 (Supplemental Figure 2). All 45 patients tested presented organ-specific autoantibodies. In total, IFN-ω autoantibodies were found most frequently (42 patients, 93%), followed by autoantibodies against 21OH (71%) and IL-22 (71%). Notably, the prostate-specific antigen TGM4 was found only in males (Supplemental Figure 2 and Supplemental Table 2). Assay of autoantibodies over time revealed a pattern dominated by stable positivity. However, 10 patients lost reactivity against 21OH during the follow-up period, and fluctuation of indices were found for several other autoantibodies such as tyrosine hydroxylase, SCC, IL-22, and GAD65 (Supplemental Figure 3).

### AIRE genotype vs phenotype

**Figure 3.**
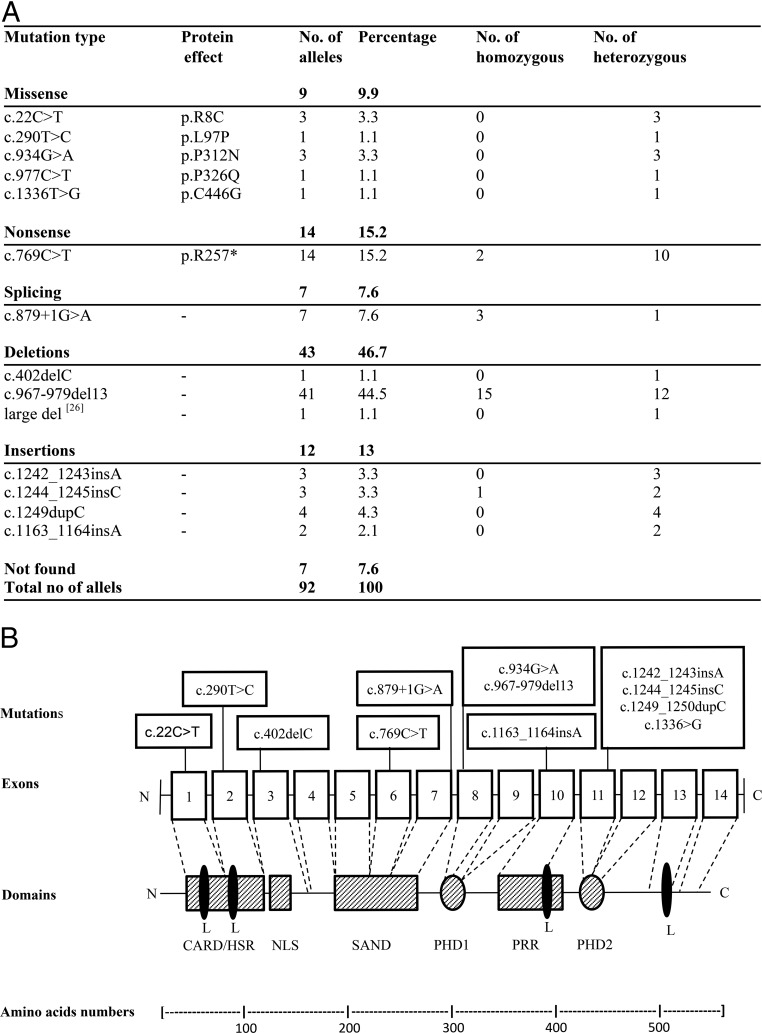
AIRE mutations in Norwegian APS1 patients. A, Overview of the identified mutations and their frequencies. B, Location of *AIRE* mutations in Norwegian APS1 patients together with a schematic representation of the AIRE protein and its functional domains. Boxes 1–14 represent the exons, and the different mutations are given in the text boxes above. CARD/HSR, Caspase recruitment domain/homodimerization domain (amino acids 1–105); NLS, nuclear localization signal (amino acids 100–189); SAND, Sp100, AIRE-1, NucP41/75 (181–280), DEAF-1; L (LXXLL), nuclear receptor-binding motifs (amino acids 7–11, 63–67, 414–418, 516–520); PHD, plant homeodomain type zinc fingers (amino acids 296–343 and 434–475); PRR, proline-rich region (amino acids 350–430).

We detected *AIRE* mutations in 44 patients. Two had no mutations or copy number variations. Six deceased patients were not tested, but genotypes could be deduced based on their siblings. The most common mutation was c.967_979del13 found in 45% of the alleles, followed by c.769C>T, and c.879+1G>A (Figure 3 and Supplemental Table 2). We then grouped them according to genotype, namely patients homozygous for missense mutations (genotype 1; seven patients), patients homozygous for mutations giving a truncated protein (genotype 2; 32 patients), and patients with one missense mutation and one mutation giving a truncated protein (genotype 3; five patients). The splicing mutations were included in group 1. The two patients without mutations (patients 49 and 50) were excluded. The median number of disease manifestations was five in all groups. However, patients with genotype 1 had a later disease onset (median age, 19 years) than genotypes 2 and 3 (median age, 7 and 11 years, respectively). Among patients with genotype 2, 90% had CMC, and 75% had PAI. All patients with asplenia had genotype 2. Three patients homozygous and one heterozygous for the splicing mutation c.879+1C>G (patients 29, 30, 31, and 48) presented a mild phenotype with late disease onset (Figure 3 and Supplemental Table 2).

### Immunotype vs phenotype

**Figure 4.**
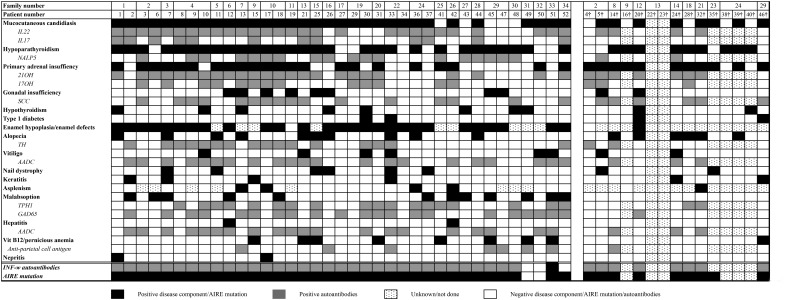
Heat map of clinical manifestations and autoantibodies in Norwegian APS1 patients. Family number and patient number are given in the first two rows. A black square represents a disease manifestation, and a gray square represents positive autoantibodies. If the square is dotted, the examination/analyses are not performed; a white square represents negative disease component/AIRE mutation/autoantibodies that are tested for.

Seventeen (49%) of the patients with hypoparathyroidism had autoantibodies against NACHT leucine-rich-repeat protein 5. Among patients with PAI, 93% had autoantibodies against 21OH, 63% against SCC, and 43% against 17OH. All of the patients with autoantibodies against 17OH also had autoantibodies against 21OH. Both autoantibodies against 21OH and SCC correlated significantly to PAI (*P* < .001 and *P* = .002, respectively). Thirty patients with CMC (81%) had autoantibodies against IL-22, giving a significant correlation (*P* = .004). Autoantibodies against GAD65 were found in 22 patients, including three patients with diabetes mellitus type 1. Seven patients with vitiligo had autoantibodies against AADC (88%), proving a significant correlation (*P* = .047). Figure 4 presents a heat map with clinical manifestations and previously reported correlated autoantibodies grouped together.

### HLA vs phenotype

The HLA class II genotype stratified to the risk of PAI is given in Supplemental Table 2. However, 10 patients with PAI had HLA class II alleles known to be protective in the general population. We also found significantly more alopecia in this group compared to the rest of the cohort. None of the APS1 patients with PAI carried the high-risk HLA class II haplotype DR3-DQ2/DR4.4-DQ8 (27).

## Discussion

This longitudinal follow-up of the 52 Norwegian APS1 patients demonstrates the clinical variability from very mild to severe disease, occurrence of new components over time, and an overall increased mortality from adrenal crisis and cancer. Dental examination revealed that enamel hypoplasia was present in most patients and, together with CMC and hypoparathyroidism, is one of the three most common manifestations. Longitudinal data have previously been presented from the Finnish, Sicilian, and Sardinian patient cohorts (with 91, 15, and 22 patients, respectively) (21–23), which all represent populations with strong founder effects (21, 23). The Norwegian population displays a greater genetic heterogeneity and may therefore be more representative for the situation in most countries.

The clinical variation and rarity of APS1 makes the syndrome hard to recognize. Since our latest report in 2007, only one child has been diagnosed, which is unexpected because we estimate that at least one child every other year is born with APS1 in Norway (3). The reason is unclear, but most likely disease components are being recognized and treated, whereas the syndrome goes undiagnosed. Alternatively, patients die during childhood without diagnosis, as indeed was observed in seven of our patients. This underscores the importance of increased awareness and early diagnosis. APS1 should be considered in all patients presenting one of the major clinical manifestations, especially when it presents in childhood. Moreover, when a new patient is diagnosed, all siblings should be offered genetic counseling.

Some variation in the frequency of disease components occurs among APS1 cohorts. We report several with hypothyroidism, which is also found in Apulian APS1 patients (28). However, hypothyroidism was not seen in the Sardinian patients (23), although late onset cannot be excluded (2). Although autoimmune cause was not proved, it is probably caused by autoimmunity given the propensity for autoimmunity in APS1. Furthermore, autoimmune hepatitis was described as a serious and early feature in 27% of the Sardinian patients (23), whereas we found hepatitis in only two (4%). In a study of 23 Persian Jews (11), 22 presented with hypoparathyroidism, and only four patients had oral CMC. The Sardinian patients displayed the most severe phenotype, with a mean of seven disease manifestations per patient and early disease onset (23). Besides the different *AIRE* genotypes, other immune genes might potentially affect the phenotype (29), and environmental factors and varying practices among clinicians may also have influence.

Poorly treated or undiagnosed endocrinopathies as part of APS1, especially PAI, hypoparathyroidism, and diabetes mellitus type 1, can be fatal. Adherence to therapy, especially in teenage patients, is challenging. An increased death risk and altered cancer incidence pattern have been described (4). Identifying risk factors for malignancies and minimizing these by treatment of CMC and avoidance of smoking are probably important (21). In addition, pneumococcal vaccination must be performed in patients with asplenia and should probably be offered to all APS1 patients.

All of the Norwegian patients had organ-specific autoantibodies. Typically, the presence of autoantibodies correlates to clinical manifestations but may appear years before the corresponding clinical manifestation (3, 14, 18, 19). A correlation between gonadal failure and autoantibodies against SCC is reported (14, 30), which was also found in five of the nine female patients with gonadal failure in this study. In total, SCC autoantibodies were found in 21 patients. Autoantibodies against GAD65 are normally known to correlate with diabetes mellitus type 1 (31), but this is not the case in APS1 (14). We found GAD65 autoantibodies in three of four diabetic patients and in 19 patients without diabetes. No correlation with autoantibodies against GAD65 and vitiligo or malabsorption was found, in conflict with an earlier report (14). However, autoantibodies against AADC correlated with vitiligo, and autoantibodies against 21OH and SCC correlated with PAI. Autoantibodies against the prostate-specific enzyme TGM4 were only seen in the males, consistent with a recent report (15).

We found antibodies against IFN-ω in a similar proportion to that in other APS1 cohorts (21–23). These autoantibodies are often found in the earliest samples; they persist for decades and show a high specificity for APS1 (19, 32). IFN autoantibodies can also be found in low titer in diseases causing an increased IFN production (ie, systemic lupus erythematosus, human immunodeficiency virus, and hepatitis C virus infections), as well as in myasthenia gravis (33). Two female patients did not present *AIRE* mutations or IFN-ω autoantibodies but fulfilled the clinical criteria. They may have mutations either in the regulatory parts of the *AIRE* gene or in other genes in the same pathway, or they may be phenocopies. Another patient presented all three major disease components from childhood and was compound heterozygous (c.22C>T/c.967_979del13) for two *AIRE* mutations, but autoantibodies against IFN-ω were not found.

The cohort presented here is older and has a much greater genetic heterogeneity compared to other APS1 cohorts. Of particular interest was the c.879+1C>G splice mutation found in three homozygous patients (patients 29, 30, and 31), who all had ancestors in a particular district of Western Norway. Their phenotypes were characterized by late disease onset and generally a milder phenotype. The three patients had their first manifestation at 15, 19, and 23 years of age. The two patients with hypoparathyroidism developed hypocalcemia at 43 and 60 years of age. It is not known whether this implies that the splice defect is not complete and that some residual AIRE function is present.

We found 10 patients with PAI carrying HLA class II alleles known to protect against PAI in the general population (27). Patients in this group also had significantly more alopecia (*P* < .05). In contrast, none of the APS1 patients with PAI carried the HLA class II haplotype DR3-DQ2/DR4.4-DQ8, which is by far the strongest predisposing genetic factor for autoimmune PAI. Moreover, three out of four patients with diabetes carried the DQB1*0602 allele, which is otherwise extremely rare in type 1 diabetes. Although many of these patients are relatives, this indicates that the known risk stratification for HLA is overridden by the effect of the *AIRE* mutations, in contrast to previous findings (29).

In conclusion, the increasing knowledge about clinical variation, *AIRE*, and autoimmunity seems to expand and redefine APS1. However, the diagnosis should be considered in all patients presenting one of the major clinical manifestations, especially when it first presents in childhood. Nonendocrine components such as enamel hypoplasia and CMC are common and should trigger further diagnostic workup. Autoantibodies against IFN-ω are usually present, but their absence does not exclude the diagnosis. The *AIRE* gene should be sequenced if clinical suspicion is high. When a patient is diagnosed, all siblings should be investigated because late onset is common. We recommend regular surveillance in a specialized center because it can reduce morbidity and mortality.
